# Exercise and inflammation in female survivors of breast cancer: systematic review and meta-analysis with secondary mediation analysis on body composition

**DOI:** 10.1007/s11764-025-01873-7

**Published:** 2025-07-26

**Authors:** Francesco Bettariga, Dennis R. Taaffe, Anita Borsati, Alice Avancini, Sara Pilotto, Stefano G. Lazzarini, Pedro Lopez, Luca Maestroni, Umberto Crainich, Enrico Bandini, John P. Campbell, Timothy D. Clay, Daniel A. Galvão, Robert U. Newton

**Affiliations:** 1 Exercise Medicine Research Institute, Edith Cowan University, Joondalup, Australia; 2 School of Medical and Health Sciences, Edith Cowan University, Joondalup, Australia; 3 Biomedical, Clinical and Experimental Sciences, Department of Medicine, University of Verona, Verona, Italy; 4 Department of Neurosciences, Biomedicine and Movement, University of Verona, Verona, Italy; 5 Section of Innovation Biomedicine-Oncology Area, Department of Engineering for Innovation Medicine (DIMI), University of Verona and University and Hospital Trust (AOUI) of Verona, Verona, Italy; 6 Department of Statistics and Quantitative Methods, University of Milano-Bicocca, Milan, Italy; 7 IRCCS Fondazione Don Carlo Gnocchi, Milan, Italy; 8 Pleural Medicine Unit, Institute for Respiratory Health, Perth, WA 6009 Australia; 9 Programa de Pós-Graduação Em Ciências da Saúde, Universidade de Caxias Do Sul, Caxias Do Sul, Rio Grande Do Sul, 95070-560 Brazil; 10 Grupo de Pesquisa Em Exercício Para Populações Clínicas (GPCLIN), Universidade de Caxias Do Sul, Caxias Do Sul, Rio Grande Do Sul, 95070-560 Brazil; 11 School of Science and Technology, London Sport Institute, Middlesex University, London, UK; 12 Department for Health, University of Bath, Bath, UK; 13 Department of Oncology, St John of God Subiaco Hospital, Perth, WA Australia; 14 School of Human Movement and Nutrition Sciences, University of Queensland, St Lucia, Australia

**Keywords:** Exercise, Inflammation, Breast cancer, Body composition

## Abstract

**Purpose:**

Inflammation plays a key role in breast cancer recurrence and mortality by promoting tumor progression. Exercise significantly reduces pro-inflammatory markers such as IL-6, TNF-α, and CRP, potentially reducing mortality risk. However, it is yet to be determined whether exercise-induced changes in body composition can modulate inflammatory biomarker responses in the breast cancer setting.

**Methods:**

We conducted a secondary mediation analysis based on the findings from our previously published systematic review with meta-analysis. Randomized controlled trials examining the effects of exercise on IL-1β, IL-6, IL-8, IL-10, TNF-α, and CRP and body composition and anthropometry measures such as lean mass (LM), fat mass (FM), body weight and body mass index (BMI) were included. Random-effect meta-regression models were undertaken.

**Results:**

Sixteen studies were included in the secondary analysis (n = 752). Increases in LM were significantly associated with reductions in IL-6 (*p* = 0.03) and there was a trend toward decreases in IL-8 (*p* = 0.05). In addition, there were non-significant trends suggesting that reductions in FM may be associated with decreases in IL-6 (*p* = 0.08) and TNF-α (*p* = 0.06), and that decreases in BMI may be linked to changes in IL-8 (*p* = 0.06).

**Conclusions:**

Changes in body composition following exercise were not consistently associated with inflammatory biomarker responses in breast cancer survivors with only LM significantly associated with IL-6 while a trend in LM and FM existed for some other inflammatory markers. Although these findings do not provide conclusive evidence, they highlight a possible role of exercise-induced body composition changes in mediating inflammation. Further research is needed to clarify whether targeted exercise interventions that improve body composition can contribute to reducing inflammation and, ultimately, impact breast cancer progression, recurrence, and mortality.

**Supplementary Information:**

The online version contains supplementary material available at https://doi.org/10.1007/s11764-025-01873-7.

## Introduction

Breast cancer is the most diagnosed and leading cause of cancer-related death in women worldwide [1]. Chronic inflammation plays a crucial role in breast cancer development, progression, and recurrence [2, 3]. Indeed, a persistent inflammatory state not only promotes tumor progression by influencing cell proliferation, survival, invasiveness, and metastasis [2–5], but also inhibits immune function [6]. Given that the cancer itself and the side-effects of treatments (e.g., chemotherapy) can elevate levels of inflammatory biomarkers (i.e., interleukin 6 [IL-6], tumor necrosis factor alpha [TNF-α], and C-reactive protein [CRP]) [7, 8], survivors of breast cancer are at increased risk of cancer progression, recurrence and mortality [9–11]. As a result, strategies are needed to ameliorate inflammatory status which may provide a less supportive environment for cancer progression, leading to a lower risk of recurrence and mortality in survivors of breast cancer.

Exercise is widely recognized as a key therapeutic strategy in breast cancer management [12–14]. It is well known that exercise is safe and effective before, during and after treatment, improving physical fitness, body composition (e.g., lean mass [LM] and fat mass [FM]), and fatigue while also reducing recurrence and mortality risk by ≈ 40% [12–16]. Although more research is needed, it has been proposed that exercise may modulate inflammation [17–19]. This may be partly due to skeletal muscle acting as an endocrine organ that releases anti-inflammatory cytokines at rest and in response to muscle contraction [18–21]. These myokines can exert systemic effects, including modulation of immune function, inhibition of pro-inflammatory cytokines, and promotion of an anti-inflammatory milieu [22, 23]. For example, exercise-induced IL-6 stimulates the production of anti-inflammatory cytokines such as IL-10 and inhibits TNF-α, a key driver of chronic inflammation linked to cancer progression [24]. Further, myokines can improve insulin sensitivity, reduce adipose tissue inflammation, and enhance the recruitment and activation of natural killer cells and cytotoxic T-cells, which are critical for immune surveillance and tumor suppression [19, 22, 23, 25]. In addition, it appears that muscle mass levels may influence the magnitude and profile of myokine release, potentially contributing to a less favorable environment for cancer progression [18]. In this regard, we recently examined the effects of exercise on several inflammatory biomarkers in survivors of breast cancer [26]. We observed that exercise programs, specifically combined exercise (i.e., resistance training [RT] and aerobic training [AT]), effectively reduced the levels of some pro-inflammatory biomarkers, such as IL-6, TNF-α, and CRP, while other biomarkers (e.g., IL-1β, IL-8, IL-10) were not significantly altered [26].

In the past two decades, the role of body composition in relation to mortality risk has gained substantial attention [27–29]. Several studies observed that low skeletal muscle mass as well as increased adipose tissue are strong predictors of mortality risk in breast cancer [27–29]. However, to date, no studies have examined whether exercise-induced changes in body composition can modulate inflammatory biomarker responses in the breast cancer setting. Indeed, findings from a mediation analysis may enhance our understanding of how exercise and its effects on body composition may, consequently, impact inflammatory biomarkers. Thus, the aim of the present exploratory secondary analysis was to investigate the role of exercise-induced changes in body composition on biomarkers of inflammation (i.e., IL-1β, IL-6, IL-8, IL-10, TNF-α, and CRP) in survivors of breast cancer.

## Methods

### Search strategy, eligibility criteria, and study selection procedure

The present study is a follow-up analysis of our previous systematic review and meta-analysis examining the effects of exercise on inflammation in female survivors of nonmetastatic breast cancer [26]. The procedures undertaken in the present study were reported in accordance with the Cochrane Back Review Group [30], and the Preferred Reporting Items for Systematic Reviews and Meta-Analyses statement [31, 32]. This systematic review was prospectively registered at the International Prospective Register of Systematic Reviews (PROSPERO identifier: CRD42024575738). Briefly, a systematic search was conducted in PubMed, CINAHL, Embase, SPORTDiscus (via EBSCOhost), and Cochrane Library (CENTRAL). The full search strategies are reported in the supplementary materials (Supplementary Table 1) [26].

For this secondary mediation analysis, we have included only randomised clinical trials assessing the effects of exercise on IL-1β, IL-6, IL-8, IL-10, TNF-α and CRP that also examined body composition and anthropometry measures such as LM, FM, body weight and body mass index (BMI). The investigation of body composition and anthropometry measures was not originally planned and this deviation from the original protocol is characterised as an *addition*. The systematic search was conducted from inception to August 2024. Further information on the methods and procedures as well as the primary results have been reported elsewhere [26].

### Statistical analysis

A three-level mixed-effects meta-analysis with study included as a random effect was performed to examine the effects of exercise training on biomarkers of inflammation and body composition and anthropometry measures [33]. Cluster robust point estimates and 95% CI were weighted by inverse sampling variance to account for the within- and between-study variance (τ^2^). Statistical heterogeneity was assessed using the Cochran Q test. High heterogeneity was defined by a threshold *p*-value of 0.1 or I^2^ values greater than 50%. The pooled effects estimated were expressed as standardised mean difference (SMD). Statistical significance was set at a *p* value < 0.05. SMD values of 0.0 to ≤ 0.5 indicate small, 0.51 to 0.79, moderate, and ≥ 0.8, large effects [34]. In addition, we considered results approaching statistical significance (*p* value ranging from 0.05 to 0.15) when SMD was above 0.50, with results reported as trend [35].

To assess the potential mediation effect of changes in body composition and anthropometry measures on changes in inflammation biomarkers, a series of random-effect meta-regression models were undertaken treating the body composition and anthropometry effect sizes as predictor variables and inflammation biomarkers as the outcome variables, when at least two studies were found. These association analyses examined if exercise-induced changes in LM, FM, body weight, and BMI were related to changes in IL-1β, IL-6, IL-8, IL-10, TNF-α, and CRP.

## Results

**Fig. 1:**
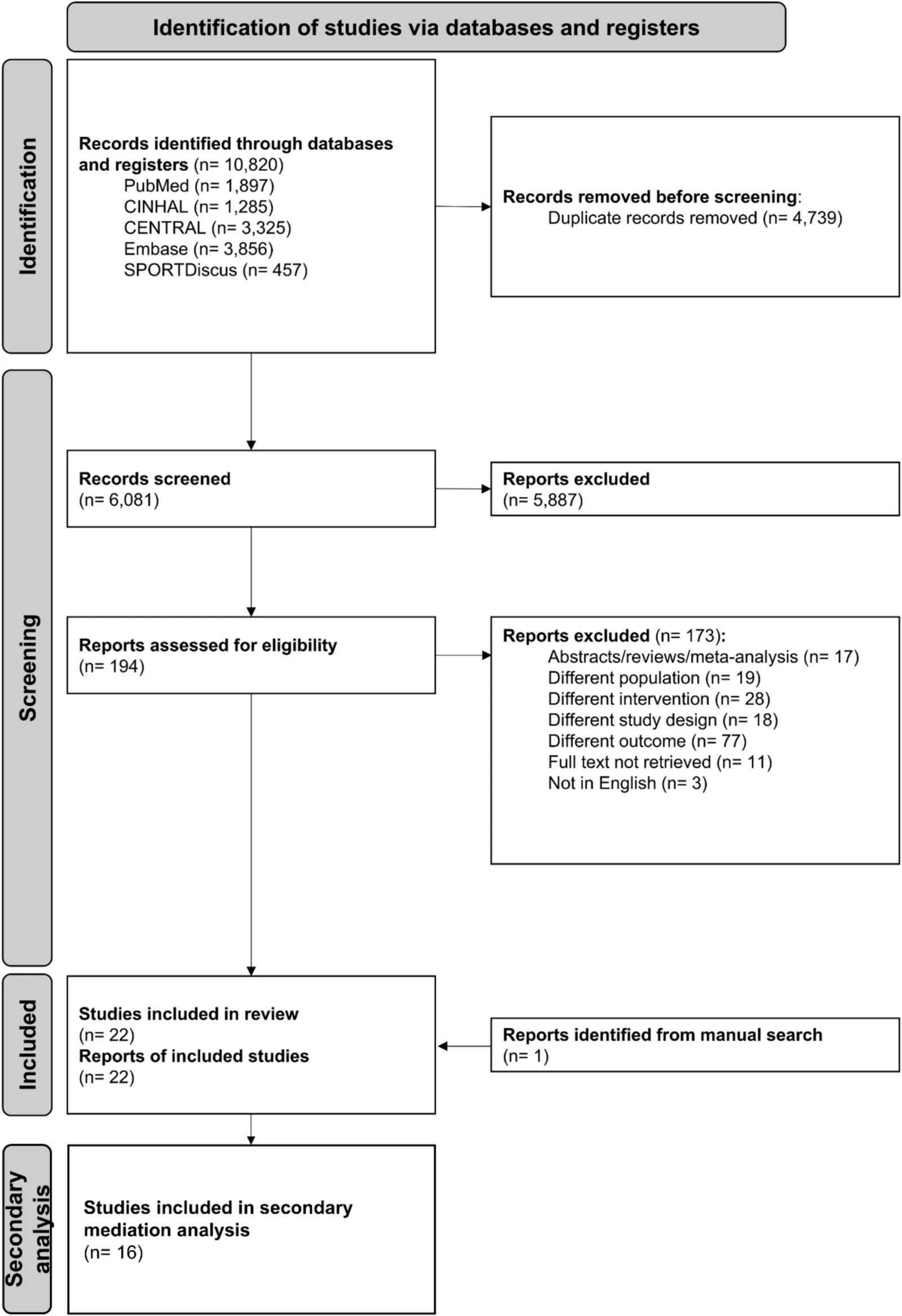
Flow chart of study selection process

The number of studies retrieved, screened, and included from our search have been previously reported [26]. From the 22 articles included in our previous review, 16 met the inclusion criteria for the mediation analysis on IL-6, IL-8, IL-10, TNF-α, and CRP [36–51], while no studies were found for IL-1β (Fig. 1). In this regard, we performed the SMD on biomarkers based on the included studies in this analysis.

### Participants and intervention characteristics

Seven-hundred and fifty-two survivors of breast cancer, median age of 53.3 years (interquartile range [IQR]: 49.1, 58.1) and BMI of 28.6 kg/m^2^ (IQR: 26.4, 32.6), participated in the included studies [36–51]. Characteristics of the individual studies are reported in Supplementary Table 2. Participants in the included studies were diagnosed with breast cancer stage I-III. Four exercise arms employed RT [40, 46–48], five AT [41, 43, 44], and nine combined RT and AT [36–39, 42, 45, 49–51], with duration ranging from 8–52 weeks and frequency 1–6 days per week. The overall risk of bias was moderate [26].

### Body composition and anthropometry

There was a trend toward decrease in BMI (SMD = −0.13, *p* = 0.07) and body weight (SMD = −0.32, *p* = 0.06) that approached statistical significance in favour of exercise compared to control groups. In addition, there was a significant small increase in LM in favour of exercise compared to control groups (SMD = 0.32, *p* = 0.02) with no significant change for FM (Table 1).

**Table 1:** Effect of exercise training on biomarkers of inflammation and body composition

			Random-Effect Meta-Analysis			Heterogeneity		
	***K***	***Number of ES***	***SMD***	***95% CI***	***P***	***Q***	***I***^***2***^	***P***
IL-6	10	12	−0.89	−2.08 to 0.30	0.13	59.3	86.4	0
IL-8	6	7	−0.84	−2.66 to 0.98	0.29	44.9	87.8	0
IL-10	8	10	0.08	−0.29 to 0.46	0.61	4.7	0	0.86
TNF-α	9	11	−0.45	−1.00 to 0.10	0.10	14.1	38.6	0.17
CRP	9	9	−0.64	−1.58 to 0.30	0.15	36.1	39.2	0
BMI	11	12	−0.13	−0.27 to 0.01	0.07	1.3	0	1
Body weight	10	12	−0.32	−0.65 to 0.02	0.06	11.7	0	0.38
Lean mass	8	11	0.32	0.07 to 0.57	0.02	3.5	0	0.96
Fat mass	6	8	−0.73	−1.72 to 0.26	0.12	15.8	65	0.02

*K* = number of studies; *ES* = effect size; *SMD* = standardized mean difference; *CI* = confidence interval; *Q* = Cochran’s Q test of heterogeneity;* I*^2^ = percentage of variation across studies that is due to heterogeneity; *P* = p-value

### Association of changes in body composition and anthropometry with changes in IL-6

A trend toward a decrease in IL-6 was observed in favour of exercise compared to control groups (SMD = −0.89 SMD, *p* = 0.13) (Table 1). In the meta-regression, increases in LM were significantly associated with reductions in IL-6 (estimate = −2.74 ± 0.61, *p* = 0.03). The association between reductions in FM and reductions in IL-6 approached statistical significance (estimate = 1.08 ± 0.31, *p* = 0.08), while changes in BMI and body weight did not mediate changes in IL-6 (Table 2).

**Table 2:** Mediation analysis between biomarkers of inflammation and body composition changes after exercise programs

Biomarkers	Estimate			
	BMI	Body weight	Lean mass	Fat mass
IL-6	6.55 ± 2.57	0.73 ± 0.37	−2.74 ± 0.61 *	1.08 ± 0.31 ^
IL-8	10.08 ± 2.23 ^	0.53 ± 0.55	−4.61 ± 1.13 ^	0.56 ± 0.65
IL-10	−0.37 ± 1.42	−0.37 ± 0.45	−0.56 ± 1.75	−0.02 ± 0.37
TNF-α	3.41 ± 1.35	−0.01 ± 0.87	−1.26 ± 1.03	1.18 ± 0.28 ^
CRP	1.96 ± 2.65	0.61 ± 1.25	−3.19 ± 1.53	0.74 ± 0.82

^*^ = *p* < 0.05; ^ =  ≥ 0.5 *p* < 0.15

### Association of changes in body composition and anthropometry with changes in IL-8

There was no significant change in IL-8 between exercise and control groups (SMD = −0.84, *p* = 0.29) (Table 1). In the meta-regression, the association between reductions in BMI and reductions in IL-8 (estimate = 10.08 ± 2.23, *p* = 0.06), as well as increases in LM and reductions in IL-8 (estimate = −4.61 ± 1.13, *p* = 0.05) approached statistical significance, while changes in body weight and FM did not mediate changes in IL-8 (Table 2).

### Association of changes in body composition and anthropometry with changes in IL-10

There was no significant change in IL-10 between exercise and control groups (SMD = 0.08, *p* = 0.61) (Table 1). In the meta-regression, there were no significant associations between changes in BMI, body weight, LM, and FM and changes in IL-10 (Table 2).

### Association of changes in body composition and anthropometry with changes in TNF-α

A trend toward a decrease in TNF-α was observed in favour of exercise compared to control groups (SMD = −0.45, *p* = 0.10) (Table 1). In the meta-regression, the association between reductions in FM and reductions in TNF-α approached statistical significance (estimate = −1.18 ± 0.28, *p* = 0.06), while changes in BMI, body weight, and LM did not mediate changes in TNF-α (Table 2).

### Association of changes in body composition and anthropometry with changes in CRP

A trend toward a decrease in CRP was observed in favour of exercise compared to control groups (SMD = −0.64, *p* = 0.15) (Table 1). In the meta-regression, there were no significant associations between changes in BMI, body weight, LM, and FM and changes in CRP (Table 2).

## Discussion

In this exploratory mediation analysis, we investigated whether exercise-induced changes in body composition and anthropometry, mediated changes in inflammatory biomarkers in survivors of breast cancer. There were two important findings. First, increases in LM were associated with specific changes in IL-6 and showed a trend toward decreases in IL-8, while reductions in FM were associated with a trend toward reductions in IL-6 and TNF-α. Second, there was a trend for decreases in BMI to mediate changes in IL-8. These findings provide initial evidence of the potential role of body composition in modulating inflammation in patients with breast cancer; however, given that most associations were not statistically significant, these results should be interpreted with caution.

BMI, as a general indicator of body anthropometry, has been associated with inflammatory responses, although it does not distinguish between LM and FM [52, 53]. The relationship between reductions in BMI and inflammatory biomarkers may reflect that exercise-induced changes in body weight may impact the levels of some pro-inflammatory markers (i.e., IL-8). Similarly, we observed that reducing FM may attenuate the release of IL-6 and TNF-α. While these associations did not reach statistical significance, they align with existing evidence linking adipose tissue as a major source of pro-inflammatory biomarkers which, in turn, contribute to persistent chronic inflammation [52, 53]. This underlines the importance of targeting FM reduction in exercise programs or multimodal programs comprising exercise and nutrition, although further research is needed to confirm these effects [54]. Conversely, increases in LM induced significant reductions in IL-6 and a trend toward decrease in IL-8. The underlying reasons may be due to the role of skeletal muscle in secreting myokines which have anti-inflammatory effects [18–21]. Indeed, myokines (e.g., IL-6, IL-10, irisin, and SPARC), help regulate immune function by inhibiting pro-inflammatory cytokines such as TNF-α and IL-1β, reducing macrophage infiltration in adipose tissue, and promoting a shift from M1 to M2 macrophage phenotypes [20, 55]. Furthermore, myokines can modulate systemic inflammation through pathways such as NF-κB, JAK/STAT, and AMPK, all of which are involved in inflammation, metabolism, and immune responses [22, 56, 57]. These mechanisms may help explain the observed associations between increased muscle mass and reductions in circulating inflammatory markers. This may indicate that targeted exercise increasing skeletal muscle mass may drive lower levels of pro-inflammatory biomarkers. Interestingly, it should be noted that changes in IL-10 and CRP were not associated with body composition changes. Although speculative, this may be related to the half-life of these biomarkers, the heterogeneity as biomarkers (i.e., involved in several sources of inflammation), as well as to the individual response of each marker to exercise, although more research is needed [55, 58, 59]. Taken together, findings from this analysis contribute to our understanding of how exercise programs may influence biomarkers of inflammation and the potential mediating role of body composition, although further research is required.

Given the well-established association between body composition and mortality risk in survivors of breast cancer [27–29], such findings may be of clinical relevance. Excess adiposity and low skeletal muscle mass are strong predictors of mortality [27–29], highlighting the need for targeted interventions to promote favourable body composition changes. From a clinical perspective, we highlight the need for tailored exercise prescription designed to reduce FM while enhancing LM. Beyond this general recommendation, it would seem important to assess muscle and fat mass in survivors of breast cancer, preferably via dual energy x-ray absorptiometry (as it provides a precise and reliable measurement of body composition, including lean and fat tissue distribution), and tailor exercise prescription to address the greater imbalance as priority [60]. For example, a patient with high fat mass and adequate muscle mass requires emphasis on diet modification and high-volume AT with some RT to prevent muscle decline while driving fat loss [12]. A patient with low muscle mass requires high volume RT to drive muscle hypertrophy, minimal AT, and potentially protein supplementation [12]. Given that chronic inflammation contributes to breast cancer progression, recurrence, and mortality [9–11], strategies aimed at modulating inflammatory responses should be a key focus in survivors of breast cancer. Thus, exercise-induced improvements in body composition showed trends toward reductions in inflammation [61, 62], which may have implications for lowering the risk of disease progression and recurrence. However, these findings were not consistently statistically significant, and the underlying mechanisms remain to be fully elucidated. Therefore, further research is warranted to confirm these potential effects.

### Limitations

This secondary analysis has several limitations that should be acknowledged. Findings reaching statistical significance or indicating a possible trend were limited, and thus interpretations should be made cautiously. This may be partly due to variability in biomarker responses, differences in assay sensitivity, and the complex nature of inflammatory markers**.** These factors can contribute to inconsistent findings across studies and may have limited the ability to detect clear associations between changes in body composition and inflammatory marker responses. Addressing these limitations will be essential to strengthen the understanding of exercise’s role in modulating inflammation and cancer outcomes.

## Conclusion

Changes in body composition following an exercise program may influence inflammatory biomarker responses in survivors of breast cancer. Specifically, reductions in BMI and FM were associated with a trend toward lower levels of IL-6, IL-8, and TNF-α, while increases in LM were associated with significant reductions in IL-6 and a trend toward lower IL-8 levels, although most of these changes did not reach statistical significance. From these results we highlight the potential role of exercise-induced body composition to mediate inflammation, which is a key factor in cancer progression and mortality. Given the clinical relevance of inflammation as well as body composition in breast cancer, targeted exercise interventions that optimize FM loss while also enhancing LM, prioritized according to balance between the two, may represent a promising strategy to potentially reduce the risk of breast cancer progression, recurrence, and mortality. However, further research is needed to confirm these effects.

## Supplementary Information

Below is the link to the electronic supplementary material.

Supplementary file1 (DOCX 32 KB)

## Data Availability

No datasets were generated or analysed during the current study.

## References

[1] Bray F, et al. Global cancer statistics 2022: GLOBOCAN estimates of incidence and mortality worldwide for 36 cancers in 185 countries. CA Cancer J Clin. 2024;74(3):229–63. 10.3322/caac.21834 38572751

[2] Greten FR, Grivennikov SI. Inflammation and cancer: triggers, mechanisms, and consequences. Immunity. 2019;51(1):27–41. 10.1016/j.immuni.2019.06.025 PMC6831096 31315034

[3] Vendramini-Costa DB, Carvalho JE. Molecular link mechanisms between inflammation and cancer. Curr Pharm Des. 2012;18(26):3831–52. 10.2174/138161212802083707 22632748

[4] Korkaya H, Liu S, Wicha MS. Breast cancer stem cells, cytokine networks, and the tumor microenvironment. J Clin Invest. 2011;121(10):3804–9. 10.1172/JCI57099 PMC3223613 21965337

[5] Zhao H, et al. Inflammation and tumor progression: signaling pathways and targeted intervention. Signal Transduct Target Ther. 2021;6(1):263. 10.1038/s41392-021-00658-5 PMC8273155 34248142

[6] Blank CU et al. CANCER IMMUNOLOGY. The "cancer immunogram". Science. 2016;352(6286): p. 658–60. 10.1126/science.aaf2834 27151852

[7] Mills RC 3rd. Breast cancer survivors, common markers of inflammation, and exercise: a narrative review. Breast Cancer (Auckl). 2017;11:1178223417743976. 10.1177/1178223417743976 PMC5802700 29434469

[8] Bower JE et al. Acute and Chronic Effects of Adjuvant Therapy on Inflammatory Markers in Breast Cancer Patients. JNCI Cancer Spectr. 2022;6(4). 10.1093/jncics/pkac052 PMC9420043 35900175

[9] Kolberg H-C, et al. The role of C-reactive protein (CRP) as a prognostic biomarker in patients with early breast cancer (EBC) treated with neoadjuvant chemotherapy (NACT). J Clin Oncol. 2021;39(15_suppl):e12545–e12545.

[10] Chen J, et al. IL-6: the link between inflammation, immunity and breast cancer. Front Oncol. 2022;12:903800. 10.3389/fonc.2022.903800 PMC9341216 35924148

[11] Bachelot T, et al. Prognostic value of serum levels of interleukin 6 and of serum and plasma levels of vascular endothelial growth factor in hormone-refractory metastatic breast cancer patients. Br J Cancer. 2003;88(11):1721–6. 10.1038/sj.bjc.6600956 PMC2377148 12771987

[12] Hayes SC, et al. The Exercise and Sports Science Australia position statement: exercise medicine in cancer management. J Sci Med Sport. 2019;22(11):1175–99. 10.1016/j.jsams.2019.05.003 31277921

[13] Campbell KL, et al. Exercise guidelines for cancer survivors: consensus statement from international multidisciplinary roundtable. Med Sci Sports Exerc. 2019;51(11):2375–90. 10.1249/MSS.0000000000002116 PMC8576825 31626055

[14] Schmitz KH, et al. Exercise is medicine in oncology: engaging clinicians to help patients move through cancer. CA Cancer J Clin. 2019;69(6):468–84. 10.3322/caac.21579 PMC7896280 31617590

[15] Bettariga F, et al. Association of muscle strength and cardiorespiratory fitness with all-cause and cancer-specific mortality in patients diagnosed with cancer: a systematic review with meta-analysis. Br J Sports Med. 2025;59(10):722–32. 10.1136/bjsports-2024-108671 39837589

[16] Bettariga F, et al. Effects of resistance training vs high intensity interval training on body composition, muscle strength, cardiorespiratory fitness, and quality of life in survivors of breast cancer: a randomized trial. Breast Cancer Res Treat. 2025;210(2):261–70. 10.1007/s10549-024-07559-5 39557768

[17] Meneses-Echávez JF, et al. The effect of exercise training on mediators of inflammation in breast cancer survivors: a systematic review with meta-analysis. Cancer Epidemiol Biomarkers Prev. 2016;25(7):1009–17. 10.1158/1055-9965.EPI-15-1061 27197276

[18] Bettariga F, et al. Effects of short- and long-term exercise training on cancer cells in vitro: insights into the mechanistic associations. J Sport Health Sci. 2025;14:100994. 10.1016/j.jshs.2024.100994 PMC11863286 39370102

[19] Bettariga F, et al. Suppressive effects of exercise-conditioned serum on cancer cells: a narrative review of the influence of exercise mode, volume, and intensity. J Sport Health Sci. 2024;13(4):484–98. 10.1016/j.jshs.2023.12.001 PMC11184317 38081360

[20] Pedersen BK, Febbraio MA. Muscle as an endocrine organ: focus on muscle-derived interleukin-6. Physiol Rev. 2008;88(4):1379–406. 10.1152/physrev.90100.2007 18923185

[21] Bettariga F, et al. Exercise training mode effects on myokine expression in healthy adults: a systematic review with meta-analysis. J Sport Health Sci. 2024;13(6):764–79. 10.1016/j.jshs.2024.04.005 PMC11336361 38604409

[22] Fiuza-Luces C, et al. The effect of physical exercise on anticancer immunity. Nat Rev Immunol. 2024;24(4):282–93. 10.1038/s41577-023-00943-0 37794239

[23] Severinsen MCK, Pedersen BK. Muscle-organ crosstalk: the emerging roles of myokines. Endocr Rev. 2020;41(4):594–609. 10.1210/endrev/bnaa016 PMC7288608 32393961

[24] Petersen AM, Pedersen BK. The role of IL-6 in mediating the anti-inflammatory effects of exercise. J Physiol Pharmacol. 2006;57(Suppl 10):43–51. 17242490

[25] Bettariga F et al. Acute and chronic exercise-conditioned serum effects on cancer cells in vitro: a systematic review. Eur J Cancer Prev. 2025; p. 10.1097/CEJ.0000000000000968. 41889110

[26] Bettariga F et al. Effects of exercise on inflammation in female survivors of nonmetastatic breast cancer: a systematic review and meta-analysis. JNCI: J Nat Cancer Institute. 2025. 10.1093/jnci/djaf062 PMC12505137 40112254

[27] Caan BJ, et al. Association of muscle and adiposity measured by computed tomography with survival in patients with nonmetastatic breast cancer. JAMA Oncol. 2018;4(6):798–804. 10.1001/jamaoncol.2018.0137 PMC6584322 29621380

[28] Caan BJ, et al. Post-diagnosis weight gain and breast cancer recurrence in women with early stage breast cancer. Breast Cancer Res Treat. 2006;99(1):47–57. 10.1007/s10549-006-9179-y 16541317

[29] Kang H, et al. Prognostic value of body composition measures in breast cancer patients treated with chemotherapy. Sci Rep. 2024;14(1):23309. 10.1038/s41598-024-74060-w PMC11458607 39375403

[30] Furlan AD et al. 2009 updated method guidelines for systematic reviews in the Cochrane Back Review Group. Spine (Phila Pa 1976). 2009;34(18):1929–41. 10.1097/BRS.0b013e3181b1c99f 19680101

[31] Page MJ, et al. Mapping of reporting guidance for systematic reviews and meta-analyses generated a comprehensive item bank for future reporting guidelines. J Clin Epidemiol. 2020;118:60–8. 10.1016/j.jclinepi.2019.11.010 31740319

[32] Page MJ, et al. The PRISMA 2020 statement: an updated guideline for reporting systematic reviews. BMJ. 2021;372: n71. 10.1136/bmj.n71 PMC8005924 33782057

[33] Cheung MW. Modeling dependent effect sizes with three-level meta-analyses: a structural equation modeling approach. Psychol Methods. 2014;19(2):211–29. 10.1037/a0032968 23834422

[34] Cohen J. Statistical power analysis. Curr Dir Psychol Sci. 1992;1(3):98–101.

[35] Sullivan GM, Feinn R. Using effect size-or why the P value is not enough. J Grad Med Educ. 2012;4(3):279–82. 10.4300/JGME-D-12-00156.1 PMC3444174 23997866

[36] de Paulo TRS, et al. Effects of resistance plus aerobic training on body composition and metabolic markers in older breast cancer survivors undergoing aromatase inhibitor therapy. Exp Gerontol. 2018;111:210–7. 10.1016/j.exger.2018.07.022 30077574

[37] Dethlefsen C, et al. Exercise regulates breast cancer cell viability: systemic training adaptations versus acute exercise responses. Breast Cancer Res Treat. 2016;159(3):469–79. 10.1007/s10549-016-3970-1 27601139

[38] Dieli-Conwright CM, et al. Effects of aerobic and resistance exercise on metabolic syndrome, sarcopenic obesity, and circulating biomarkers in overweight or obese survivors of breast cancer: a randomized controlled trial. J Clin Oncol. 2018;36(9):875–83. 10.1200/JCO.2017.75.7526 PMC5858524 29356607

[39] Dieli-Conwright CM, et al. Adipose tissue inflammation in breast cancer survivors: effects of a 16-week combined aerobic and resistance exercise training intervention. Breast Cancer Res Treat. 2018;168(1):147–57. 10.1007/s10549-017-4576-y PMC6233989 29168064

[40] Hagstrom AD, et al. The effect of resistance training on markers of immune function and inflammation in previously sedentary women recovering from breast cancer: a randomized controlled trial. Breast Cancer Res Treat. 2016;155(3):471–82. 10.1007/s10549-016-3688-0 26820653

[41] Hooshmand Moghadam B et al. The Effects of High-Intensity Interval Training vs. Moderate-Intensity Continuous Training on Inflammatory Markers, Body Composition, and Physical Fitness in Overweight/Obese Survivors of Breast Cancer: A Randomized Controlled Clinical Trial. Cancers (Basel). 2021;13(17). 10.3390/cancers13174386 PMC8430701 34503198

[42] Hutnick NA, et al. Exercise and lymphocyte activation following chemotherapy for breast cancer. Med Sci Sports Exerc. 2005;37(11):1827–35. 10.1249/01.mss.0000175857.84936.1a 16286849

[43] Isanejad A, et al. Comparison of the effects of high-intensity interval and moderate-intensity continuous training on inflammatory markers, cardiorespiratory fitness, and quality of life in breast cancer patients. J Sport Health Sci. 2023;12(6):674–89. 10.1016/j.jshs.2023.07.001 PMC10658315 37423313

[44] Jones SB, et al. Effect of exercise on markers of inflammation in breast cancer survivors: the Yale exercise and survivorship study. Cancer Prev Res Phila. 2013;6(2):109–18. 10.1158/1940-6207.CAPR-12-0278 PMC3839104 23213072

[45] Kim TH, et al. Effects of exercise training on circulating levels of Dickkpof-1 and secreted frizzled-related protein-1 in breast cancer survivors: A pilot single-blind randomized controlled trial. PLoS ONE. 2017;12(2):e0171771. 10.1371/journal.pone.0171771 PMC5298304 28178355

[46] Lee KJ, An KO. Impact of High-Intensity Circuit Resistance Exercise on Physical Fitness, Inflammation, and Immune Cells in Female Breast Cancer Survivors: A Randomized Control Trial. Int J Environ Res Public Health. 2022;19(9). 10.3390/ijerph19095463 PMC9102474 35564858

[47] Martins FM, et al. Lower-body resistance training reduces interleukin-1β and transforming growth factor-β1 levels and fatigue and increases physical performance in breast cancer survivors. J Strength Cond Res. 2023;37(2):439–51. 10.1519/JSC.0000000000004270 36696263

[48] Robbins RN et al. Kynurenine metabolism as a mechanism to improve fatigue and physical function in postmenopausal breast cancer survivors following resistance training. J Funct Morphol Kinesiol, 2022;**7**(2). 10.3390/jfmk7020045 PMC9225420 35736016

[49] Rogers LQ, et al. Effects of a physical activity behavior change intervention on inflammation and related health outcomes in breast cancer survivors: pilot randomized trial. Integr Cancer Ther. 2013;12(4):323–35. 10.1177/1534735412449687 PMC3909487 22831916

[50] Rogers LQ, et al. Biobehavioral factors mediate exercise effects on fatigue in breast cancer survivors. Med Sci Sports Exerc. 2014;46(6):1077–88. 10.1249/MSS.0000000000000210 PMC4435796 24212124

[51] Sturgeon KM, et al. WISER survivor trial: combined effect of exercise and weight loss interventions on inflammation in breast cancer survivors. Med Sci Sports Exerc. 2023;55(2):209–15. 10.1249/MSS.0000000000003050 PMC9840668 36170550

[52] Kolb R, Zhang W. Obesity and breast cancer: a case of inflamed adipose tissue. Cancers (Basel). 2020;12(6). 10.3390/cancers12061686 PMC7352736 32630445

[53] Iyengar NM, Hudis CA, Dannenberg AJ. Obesity and inflammation: new insights into breast cancer development and progression. Am Soc Clin Oncol Educ Book. 2013;33:46–51. 10.1200/EdBook_AM.2013.33.46 PMC3897299 23714453

[54] Olateju IV, et al. A systematic review on the effectiveness of diet and exercise in the management of obesity. Diabetes Metab Syndr. 2023;17(4):102759. 10.1016/j.dsx.2023.102759 37084486

[55] Peake JM, et al. Cytokine expression and secretion by skeletal muscle cells: regulatory mechanisms and exercise effects. Exerc Immunol Rev. 2015;21:8–25. 25826432

[56] Leal LG, Lopes MA, Batista ML Jr. Physical exercise-induced myokines and muscle-adipose tissue crosstalk: a review of current knowledge and the implications for health and metabolic diseases. Front Physiol. 2018;9:1307. 10.3389/fphys.2018.01307 PMC6166321 30319436

[57] Pedersen BK, Febbraio MA. Muscles, exercise and obesity: skeletal muscle as a secretory organ. Nat Rev Endocrinol. 2012;8(8):457–65. 10.1038/nrendo.2012.49 22473333

[58] MacNeil LG, Tarnopolsky MA, Crane JD. Acute, exercise-induced alterations in cytokines and chemokines in the blood distinguish physically active and sedentary aging. J Gerontol A Biol Sci Med Sci. 2021;76(5):811–8. 10.1093/gerona/glaa310 PMC8087278 33289019

[59] Moldoveanu AI, Shephard RJ, Shek PN. The cytokine response to physical activity and training. Sports Med. 2001;31(2):115–44. 10.2165/00007256-200131020-00004 PMC7101891 11227979

[60] Shepherd JA, et al. Body composition by DXA. Bone. 2017;104:101–5. 10.1016/j.bone.2017.06.010 PMC5659281 28625918

[61] You T, et al. Effects of exercise training on chronic inflammation in obesity : current evidence and potential mechanisms. Sports Med. 2013;43(4):243–56. 10.1007/s40279-013-0023-3 23494259

[62] Wang S, et al. Effect of exercise training on body composition and inflammatory cytokine levels in overweight and obese individuals: a systematic review and network meta-analysis. Front Immunol. 2022;13:921085. 10.3389/fimmu.2022.921085 PMC9260601 35812437

