# Supplementary material for: Exercise and inflammation in female survivors of breast cancer: systematic review and meta-analysis with secondary mediation analysis on body composition
Source: J Cancer Surviv. 2025 Jul 26;20(Suppl 1):1240–7. doi: 10.1007/s11764-025-01873-7 (PMC13676545; doi:10.1007/s11764-025-01873-7)
Supplement: Supplementary file 1 [file 11764_2025_1873_MOESM1_ESM.docx]

**Supplementary Material**

**Supplementary Table 1.** Search strategy.

| **Database** | **Search strategy** |
| --- | --- |
| **PubMed** (via pubmed.ncbi.nlm.nih.gov/) | 1. "Breast Neoplasms"[Mesh] OR ((Breast[tiab] OR Mammary[tiab]) AND (Neoplas*[tiab] OR Tumor*[tiab] OR Tumour*[Tiab] OR Malignan*[Tiab] OR Cancer*[tiab] OR Carcinom*[tiab])) 2. "Exercise Therapy"[Mesh] OR "Exercise"[Mesh] OR "exercis*"[tiab] OR "Resistance Training"[Mesh] OR "Resistance Training"[tiab] OR "Strength Training"[tiab] OR "Strengthening"[tiab] OR "Exercise Program"[tiab] OR "Weight Lift*"[tiab] OR "Weight-Lift*"[tiab] OR "Weight-Bear*"[tiab] OR "Weight Bear*"[tiab] OR "Endurance Training"[Mesh] OR "Endurance training"[tiab] OR "Aerobic training"[tiab] OR "running"[tiab] OR "cycling"[tiab] OR "High-Intensity Interval Training"[Mesh] OR "Interval Training*"[tiab] OR "Intermittent Exercis*"[tiab] OR "Intermittent Training*"[tiab] OR "Continuous Exercis*"[tiab] OR "Continuous Training*"[tiab] 3. "randomized controlled trial"[pt] 4. "controlled clinical trial"[pt] 5. randomized[tiab] 6. placebo[tiab] 7. "clinical trials as topic"[mesh:noexp] 8. "randomly"[tiab] 9. "trial"[ti] 10. #3 OR #4 OR #5 OR #6 OR #7 OR #8 OR #9 11. animals [mh] NOT humans [mh] 12. #10 NOT #11 13. #1 AND #2 AND #12 |
| **Embase** (via Embase.com) | 1. 'breast cancer'/exp OR ((Breast OR Mammary) NEAR/3 (Neoplas* OR Tumor* OR Tumour* OR Malignan* OR Cancer* OR Carcinom*)):ti,ab,kw 2. 'kinesiotherapy'/exp OR 'exercise'/exp OR 'exercis*':ti,ab,kw OR 'resistance training'/exp OR 'resistance training':ti,ab,kw OR 'strength training':ti,ab,kw OR 'strengthening':ti,ab,kw OR 'exercise program':ti,ab,kw OR 'weight lift*':ti,ab,kw OR 'weight lift*':ti,ab,kw OR 'weight bear*':ti,ab,kw OR 'weight bear*':ti,ab,kw OR 'endurance training'/exp OR 'endurance training':ti,ab,kw OR 'aerobic training':ti,ab,kw OR 'running':ti,ab,kw OR 'cycling':ti,ab,kw OR 'high intensity interval training'/exp OR 'interval training*':ti,ab,kw OR 'intermittent exercis*':ti,ab,kw OR 'intermittent training*':ti,ab,kw OR 'Continuous Exercis*':ti,ab,kw OR 'Continuous Training*':ti,ab,kw 3. 'randomized controlled trial'/exp 4. 'controlled clinical trial'/de 5. random*:ti,ab,tt 6. 'randomization'/de 7. 'intermethod comparison'/de 8. placebo:ti,ab,tt 9. (compare:ti,tt OR compared:ti,tt OR comparison:ti,tt) 10. ((evaluated:ab OR evaluate:ab OR evaluating:ab OR assessed:ab OR assess:ab) AND (compare:ab OR compared:ab OR comparing:ab OR comparison:ab)) 11. (open NEXT/1 label):ti,ab,tt 12. ((double OR single OR doubly OR singly) NEXT/1 (blind OR blinded OR blindly)):ti,ab,tt 13. 'double blind procedure'/de 14. (parallel NEXT/1 group*):ti,ab,tt 15. (crossover:ti,ab,tt OR 'cross over':ti,ab,tt) 16. ((assign* OR match OR matched OR allocation) NEAR/6 (alternate OR group OR groups OR intervention OR interventions OR patient OR patients OR subject OR subjects OR participant OR participants)):ti,ab,tt 17. (assigned:ti,ab,tt OR allocated:ti,ab,tt) 18. (controlled NEAR/8 (study OR design OR trial)):ti,ab,tt 19. (volunteer:ti,ab,tt OR volunteers:ti,ab,tt) 20. 'human experiment'/de 21. Trial:ti,tt 22. #3 OR #4 OR #5 OR #6 OR #7 OR #8 OR #9 OR #10 OR #11 OR #12 OR #13 OR #14 OR #15 OR #16 OR #17 OR #18 OR #19 OR #20 OR #21 23. (((random* NEXT/1 sampl* NEAR/8 ('cross section*' OR questionnaire* OR survey OR surveys OR database or databases)):ti,ab,tt) NOT ('comparative study'/de OR 'controlled study'/de OR 'randomised controlled':ti,ab,tt OR 'randomized controlled':ti,ab,tt OR 'randomly assigned':ti,ab,tt)) 24. ('cross‐sectional study'/de NOT ('randomized controlled trial'/de OR 'controlled clinical study'/de OR 'controlled study'/de OR 'randomised controlled':ti,ab,tt OR 'randomized controlled':ti,ab,tt OR 'control group':ti,ab,tt OR 'control groups':ti,ab,tt)) 25. ('case control*':ti,ab,tt AND random*:ti,ab,tt NOT ('randomised controlled':ti,ab,tt OR 'randomized controlled':ti,ab,tt)) 26. ('systematic review':ti,tt NOT (trial:ti,tt OR study:ti,tt)) 27. (nonrandom*:ti,ab,tt NOT random*:ti,ab,tt) 28. 'random field*':ti,ab,tt 29. ('random cluster' NEAR/4 sampl*):ti,ab,tt 30. ((review:ab AND review:it) NOT trial:ti,tt) 31. ('we searched':ab AND (review:ti,tt OR review:it)) 32. 'update review':ab 33. (databases NEAR/5 searched):ab 34. ((rat:ti,tt OR rats:ti,tt OR mouse:ti,tt OR mice:ti,tt OR swine:ti,tt OR porcine:ti,tt OR murine:ti,tt OR sheep:ti,tt OR lambs:ti,tt OR pigs:ti,tt OR piglets:ti,tt OR rabbit:ti,tt OR rabbits:ti,tt OR cat:ti,tt OR cats:ti,tt OR dog:ti,tt OR dogs:ti,tt OR cattle:ti,tt OR bovine:ti,tt OR monkey:ti,tt OR monkeys:ti,tt OR trout:ti,tt OR marmoset*:ti,tt) AND 'animal experiment'/de) 35. ('animal experiment'/de NOT ('human experiment'/de OR 'human'/de)) 36. #23 OR #24 OR #25 OR #26 OR #27 OR #28 OR #29 OR #30 OR #31 OR #32 OR #33 OR #34 OR #35 37. #22 NOT #36 38. #1 AND #2 AND #37 |
| **CINAHL** (via EBSCOhost) | 1. (MH "Breast Neoplasms+") OR TI((Breast OR Mammary) N3 (Neoplas* OR Tumor* OR Tumour* OR Malignan* OR Cancer* OR Carcinom*)) OR AB((Breast OR Mammary) N3 (Neoplas* OR Tumor* OR Tumour* OR Malignan* OR Cancer* OR Carcinom*)) OR SU((Breast OR Mammary) N3 (Neoplas* OR Tumor* OR Tumour* OR Malignan* OR Cancer* OR Carcinom*)) 2. (MH "Exercise+") OR (MH "Therapeutic Exercise+") OR (MH "Resistance Training") OR (MH "Endurance Training") OR (MH "High-Intensity Interval Training") OR TI(“exercis*” OR “resistance training” OR “strength training” OR “strengthening” OR “exercise program” OR “weight lift*” OR “weight lift*” OR “weight bear*” OR “weight bear*” OR “endurance training” OR “aerobic training” OR “running” OR “cycling” OR “interval training*” OR “intermittent exercis*” OR “intermittent training*” OR "Continuous Exercis*" OR "Continuous Training*") OR AB(“exercis*” OR “resistance training” OR “strength training” OR “strengthening” OR “exercise program” OR “weight lift*” OR “weight lift*” OR “weight bear*” OR “weight bear*” OR “endurance training” OR “aerobic training” OR “running” OR “cycling” OR “interval training*” OR “intermittent exercis*” OR “intermittent training*” OR "Continuous Exercis*" OR "Continuous Training*") OR SU(“exercis*” OR “resistance training” OR “strength training” OR “strengthening” OR “exercise program” OR “weight lift*” OR “weight lift*” OR “weight bear*” OR “weight bear*” OR “endurance training” OR “aerobic training” OR “running” OR “cycling” OR “interval training*” OR “intermittent exercis*” OR “intermittent training*” OR "Continuous Exercis*" OR "Continuous Training*") 3. (MH "randomized controlled trials") 4. (MH "double‐blind studies") 5. (MH "single‐blind studies") 6. (MH "random assignment") 7. (MH "pretest‐posttest design") 8. (MH "cluster sample") 9. TI(randomised OR randomized) 10. AB(random*) 11. TI(trial) 12. (MH "sample size") AND AB(assigned OR allocated OR control) 13. (MH "placebos") 14. PT("randomized controlled trial") 15. AB(control W5 group) 16. (MH "crossover design") OR (MH "comparative studies") 17. AB(cluster W3 RCT) 18. (MH "Animals+") 19. (MH "animal studies") 20. TI(animal model*) 21. #18 OR #19 OR #20 22. (MH "human") 23. #21 NOT #22 24. #3 OR #4 OR #5 OR #6 OR #7 OR #8 OR #9 OR #10 OR #11 OR #12 OR #13 OR #14 OR #15 OR #16 OR #17 25. #24 NOT #23 26. #1 AND #2 AND #25 |
| **SPORTDiscus** (via EBSCOhost) | S1 DE "BREAST cancer" OR TI((Breast OR Mammary) N3 (Neoplas* OR Tumor* OR Tumour* OR Malignan* OR Cancer* OR Carcinom*)) OR AB((Breast OR Mammary) N3 (Neoplas* OR Tumor* OR Tumour* OR Malignan* OR Cancer* OR Carcinom*)) OR SU((Breast OR Mammary) N3 (Neoplas* OR Tumor* OR Tumour* OR Malignan* OR Cancer* OR Carcinom*))  S2 DE "EXERCISE" OR DE "EXERCISE therapy" OR DE "RESISTANCE training" OR DE "WEIGHT training" OR DE "STRENGTH training" OR DE "ANAEROBIC exercises" OR DE "AEROBIC exercises" OR DE "CIRCUIT training" OR DE "COMPOUND exercises" OR DE "RUNNING" OR TI(“exercis*” OR “resistance training” OR “strength training” OR “strengthening” OR “exercise program” OR “weight lift*” OR “weight lift*” OR “weight bear*” OR “weight bear*” OR “endurance training” OR “aerobic training” OR “running” OR “cycling” OR “interval training*” OR “intermittent exercis*” OR “intermittent training*” OR "Continuous Exercis*" OR "Continuous Training*") OR AB(“exercis*” OR “resistance training” OR “strength training” OR “strengthening” OR “exercise program” OR “weight lift*” OR “weight lift*” OR “weight bear*” OR “weight bear*” OR “endurance training” OR “aerobic training” OR “running” OR “cycling” OR “interval training*” OR “intermittent exercis*” OR “intermittent training*” OR "Continuous Exercis*" OR "Continuous Training*") OR SU(“exercis*” OR “resistance training” OR “strength training” OR “strengthening” OR “exercise program” OR “weight lift*” OR “weight lift*” OR “weight bear*” OR “weight bear*” OR “endurance training” OR “aerobic training” OR “running” OR “cycling” OR “interval training*” OR “intermittent exercis*” OR “intermittent training*” OR "Continuous Exercis*" OR "Continuous Training*")  S3 TX ( (clinic* N3 trial) or (controlled N3 trial) or (comparative N3 trial) or (placebo N3 trial) or (prospective N3 trial) or (randomi?ed N3 trial) ) or TX ( (clinic* N3 study) or (controlled N3 study) or (comparative N3 study) or (placebo N3 study) or (prospective N3 study) or (randomi?ed N3 study) )  S4 TX ( (random* N7 allot*) or (random* N7 assign*) or (random* N7 basis*) or (random* N7 divid*) or (random* N7 order*))  S5 TX ( (singl* N7 blind*) or (doubl* N7 blind*) or (trebl* N7 blind*) or (tripl* N7 blind*) ) or TX ( (singl* N7 mask*) or (doubl* N7 mask*) or (trebl* N7 mask*) or (tripl* N7 mask*) )  S6 TX (cross#over*) or TX (cross N1 over*)  S7 TX randomi?ed control* trial*  S8 TX ( (allocat* N3 condition*) or (allocat* N3 experiment*) or (allocat* N3 intervention*) or (allocat* N3 treatment*) or (allocat* N3 therap*) or (allocat* N3 control*) or (allocat* N3 group*) ) or TX ( (allot* N3 condition*) or (allot* N3 experiment*) or (allot* N3 intervention*) or (allot* N3 treatment*) or (allot* N3 therap*) or (allot* N3 control*) or (allot* N3 group*) ) or TX ( (assign* N3 condition*) or (assign* N3 experiment*) or (assign* N3 intervention*) or (assign* N3 treatment*) or (assign* N3 therap*) or (assign* N3 control*) or (assign* N3 group*) ) or TX ( (divid* N3 condition*) or (divid* N3 experiment*) or (divid* N3 intervention*) or (divid* N3 treatment*) or (divid* N3 therap*) or (divid* N3 control*) or (divid* N3 group*) )  S9 TX placebo*  S10 S3 OR S4 OR S5 OR S6 OR S7 OR S8 OR S9  S11 S1 AND S2 AND S10 |
| **CENTRAL** (via Cochrane Library) | 1. MeSH descriptor: [Breast Neoplasms] explode all trees 2. ((Breast OR Mammary) NEAR/2 (Neoplas* OR Tumor* OR Tumour* OR Malignan* OR Cancer* OR Carcinom*)):ti,ab,kw 3. #1 OR #2 4. MeSH descriptor: [Exercise Therapy] explode all trees 5. MeSH descriptor: [Exercise] explode all trees 6. MeSH descriptor: [Resistance Training] explode all trees 7. MeSH descriptor: [Endurance Training] explode all trees 8. MeSH descriptor: [High-Intensity Interval Training] explode all trees 9. (exercis* OR ((Resistance OR Strength OR Endurance OR Aerobic OR Interval OR Intermittent OR Continuous) NEAR/2 Training*) OR "Strengthening" OR (Weight NEAR/2 (Lift* OR bear*)) OR Weight-Lift* OR Weight-Bear* OR "running" OR "cycling"):ti,ab,kw 10. #4 OR #5 OR #6 OR #7 OR #8 OR #9 11. #3 AND #10 in Trials |

**Supplementary Table 2.** Characteristics of the studies included.

| **Study** | **Population** | **Study Design** | **Intervention** | **Control Group** | **Adherence** | **Blood collection** | **Markers** | **Body composition** |
| --- | --- | --- | --- | --- | --- | --- | --- | --- |
| Dieli-Conwright et al. 2018 **B**; Adipose tissue inflammation in breast cancer survivors: effects of a 16-week combined aerobic and resistance exercise training intervention | BCS I - III, **INT (n = 10)**: age = 53 ± 10 y, BMI = 33.5 ± 5.7 kg/m2, time after treatment = NR; **CON (n = 10)**: age = 55 ± 4.5 y, BMI = 33.3 ± 8.7 kg/m2, time after treatment = NR | Two-arm RCT | **Combined RT and AT** RT = 8 exercises, 10 to 15 repetitions at 60 to 80% 1RM for 3 sets; MICT = 30 to 50 min at 65 to 80% HRmax; 2 to 3 d/w for 16 w | Usual care | 97% | After 12-h rest and fasting | IL-6, IL-8, CRP | Body weight, LM, FM |
| Rogers et al. 2014 **B**; Biobehavioral factors mediate exercise effects on fatigue in breast cancer survivors | BCS I - II, **INT (n = 20)**: age = 57.2 ± 5.5 y, BMI = 29.8 ± 4.8 kg/m2, time after treatment = ≥ 4 w; **CON (n = 22)**: age = 55.2 ± 9.1 y, BMI = 32.6 ± 6.6 kg/m2, time after treatment = ≥ 4 w | Two-arm RCT | **Combined RT and AT** RT = 8 exercises with elastic band, 15 repetitions for 2 sets; MICT = 40 min at 48 to 52% HRreserve; 4 d/w for 12 w | Usual care | 92% | After 24-h rest and fasting | IL-6, IL-8, IL-10, TNF-α | BMI |
| Isanejad et al. 2023; Comparison of the effects of high-intensity interval and moderate-intensity continuous training on inflammatory markers, cardiorespiratory fitness, and quality of life in breast cancer patient | BCS I - III, **INT1 (n = 10)**: age = 44 ± 9.14 y, BMI = 26.5 ± 3.5, time after treatment = ≥ 1 m; **INT2 (n = 10)**: age = 46.3 ± 6.3 y, BMI = 28.1 ± 5.1, time after treatment = ≥ 1 m; **CON (n = 10)**: age = 44.9 ± 5 y, BMI = 27.3 ± 4.9, time after treatment = ≥ 1 m | Three-arm RCT | **INT1:** **AT** HIIT = 33 min at 60 to 90% VO2max; 3 d/w for 12 w **INT2: AT** MICT = 41 min at 60% VO2max; 3 d/w for 12 w | Usual care | 92 to 98% | After 48-h rest and fasting | IL-6, IL-10, TNF-α | BMI, body weight, LM, FM |
| Jones et al. 2013; Effect of exercise on markers of inflammation in breast cancer survivors: the Yale exercise and survivorship study | BCS I - IIIA, **INT (n = 36)**: age = 56.4 ± 9.6 y, BMI = 30.6 ± 6 kg/m2, time after treatment = ≥ 6 m; **CON (n = 32)**: age = 55.4 ± 7.6 y, BMI = 29.4 ± 7.3 kg/m2, time after treatment = ≥ 6 m | Two-arm RCT | **AT** MICT = 15 to 30 min at 50 to 80% HRmax; 5 d/w for 26 w | Usual care | 80% | After 12-h rest and fasting | IL-6, TNF-α, CRP | BMI, body weight, LM |
| Rogers et al. 2013 **A**; Effects of a physical activity behavior change intervention on inflammation and related health outcomes in breast cancer survivors: pilot randomized trial | BCS I - IIIA, **INT (n = 11)**: age = 58 ± 6.1 y, BMI = 33.9 ± 7.4 kg/m2, time after treatment = ≥ 8 w; **CON (n = 9)**: age = 53.7 ± 13.9 y, BMI = 30.3 ± 7.11 kg/m2, time after treatment = ≥ 8 w | Two-arm RCT | **Combined RT and AT** RT = 8 exercises, 20 repetitions at moderate intensity; MICT = 150 min at moderate intensity per week; 3 d/w for 12 w | Education | 73 to 100% | After 24-h rest and fasting | IL-6, IL-8, IL-10, TNF-α | BMI |
| Dieli-Conwright et al. 2018 **A**; Effects of aerobic and resistance exercise on metabolic syndrome, sarcopenic obesity, and circulating biomarkers in overweight or obese survivors of breast cancer: a randomized controlled trial | BCS I - III, **INT (n = 46)**: age = 52.8 ± 10.6 y, BMI = 33.1 ± 5.7 kg/m2, time after treatment = 1.8 m; **CON (n = 45)**: age = 53.6 ± 10.1 y, BMI = 33.4 ± 5.2 kg/m2, time after treatment = 1.2 m | Two-arm RCT | **Combined RT and AT** RT = 10 repetitions for 3 sets; MICT = 50 min at 65 to 80% HRmax; 3 d/w for 16 w | Usual care | 95% | After 12-h rest and fasting | IL-6, IL-8, TNF-α, CRP | BMI, body weight, LM, FM |
| Kim et al. 2017 **A**; Effects of exercise training on circulating levels of Dickkpof-1 and secreted frizzled-related protein-1 in breast cancer survivors: A pilot single-blind randomized controlled trial | BCS I - III, **INT (n = 11)**: age = 56 ± 6.5 y, BMI = 23.9 ± 2.7 kg/m2, time after treatment = ≥ 6 m; **CON (n = 13)**: age = 49.3 ± 4.8 y, BMI = 25 ± 4.7 kg/m2, time after treatment = ≥ 6 m | Two-arm RCT | **Combined RT and AT** RT = exercises with body weight and elastic band, 12 to 16 repetitions for 1 to 3 sets; MICT = 20 min at RPE 11 to 15; 3 d/w for 12 w | Usual care | NR | After 12-h rest and fasting | CRP | Body weight |
| Moghadam et al. 2021; The effects of high-intensity interval training vs. moderate-intensity continuous training on inflammatory markers, body composition, and physical fitness in overweight/obese survivors of breast cancer: a randomized controlled clinical trial | BCS I - III, **INT1 (n = 13)**: age = 57 ± 1 y, BMI = 28.2 ± 2.2, time after treatment = ≥ 6 m; **INT2 (n = 13)**: age = 57 ± 1 y, BMI = 28.2 ± 2.2, time after treatment = ≥ 6 m; **CON (n = 14)**: age = 57 ± 1 y, BMI = 28.2 ± 2.2, time after treatment = ≥ 6 m | Three-arm RCT | **INT1: AT** HIIT = 20 to 30 min at 90% HRmax; 3 d/w for 12 w; **INT2: AT** MICT = 20 to 30 min at 55 to 65% HRmax; 3 d/w for 12 w | Usual care | 87% | After 48-h rest and fasting | IL-6, IL-8, IL-10, TNF-α | Body weight, LM, FM |
| De Paulo et al. 2018; Effects of resistance plus aerobic training on body composition and metabolic markers in older breast cancer survivors undergoing aromatase inhibitor therapy | BCS I - IIIA undergoing hormone therapy, **INT (n = 18)**: age = 63.2 ± 7.1 y, BMI = 28.9 ± 5.2 kg/m2, time after treatment = NR; **CON (n = 18)**: age = 66.6 ± 9.6 y, BMI = 31.5 ± 6.2 kg/m2, time after treatment = NR | Two-arm RCT | **Combined RT and AT** RT = 8 exercises, 8 to 20 repetitions at 55 to 75% 1RM for 1 to 3 sets; MICT = 20 to 30 min at 50 to 80% HRmax; 3 d/w for 36 w | Stretching | 78 to 94% | After 12-h rest and fasting | CRP | Body weight, LM, FM |
| Hutnick et al. 2005; Exercise and lymphocyte activation following chemotherapy for breast cancer | BCS I - III, **INT (n = 16)**: age = 48.5 ± 10.6 y, BMI = 26.67 ± 5.35 kg/m2, time after treatment = ≥ 2 w; **CON (n = 12)**: age = 52.3 ± 9.2 y, BMI = 26.63 ± 4.13 kg/m2, time after treatment = ≥ 2 w | Two-arm RCT | **Combined RT and AT** RT = 4 exercises with elastic bands, 8 to 12 repetitions for 1 to 3 sets; MICT = 10 to 20 min at 60 to 75% functional capacity; 3 d/w for 26 w | Usual care | 76% | After 48-h rest and fasting | IL-6 | BMI, body weight |
| Dethlefsen et al. 2016; Exercise regulates breast cancer cell viability: systemic training adaptations versus acute exercise responses | BCS I - III, **INT (n = 37)**: age = 46 ± 9.6 y, BMI = 24 ± 3.8, time after treatment = 2 m; **CON (n = 37)**: age = 48.2 ± 7.8 y, BMI = 24.8 ± 3.7, time after treatment = 2 m | Two-arm RCT | **Combined RT and AT** RT = 8 to 10 repetitions at 70 to 90% 1RM for 3 sets; HIIT = 30 min at 80 to 90% HRmax; 1 d/w for 26 w | Education | 66% | After 24-h rest and fasting | IL-6, IL-8, IL-10, TNF-α | BMI, body weight, LM |
| Lee et al. 2022; Impact of high-intensity circuit resistance exercise on physical fitness, inflammation, and immune cells in female breast cancer survivors: a randomized control trial | BCS I - IIIA, **INT (n = 15)**: age = 54.7 ± 5.1 y, BMI = 23.6, time after treatment = ≥ 24 m; **CON (n = 15)**: age = 55.4 ± 4.3 y, BMI = 22.8, time after treatment = ≥ 24 m | Two-arm RCT | **RT** RT = 8 exercises, 8 to 16 repetitions at 40 to 80% 1RM for 3 to 4 sets; 2 to 3 d/w for 12 w | Usual care | NR | After 12-h rest and fasting | CRP | BMI, LM |
| Robbins et al. 2022; Kynurenine metabolism as a mechanism to improve fatigue and physical function in postmenopausal breast cancer survivors following resistance training | BCS I - III, **INT (n = 22)**: age = 61.6 ± 1.5 y, BMI = 32.7 ± 1.4, time after treatment = ≥ 6 m; **CON (n = 10)**: age = 67.2 ± 1.2 y, BMI = 28.9 ± 4.5, time after treatment = ≥ 6 m | Two-arm RCT | **RT** RT = 7 exercises, 15 to 20 repetitions to exhaustion for 3 sets; 3 d/w for 12 w | Cognitive | 86% | After 48-h rest and fasting | CRP | BMI |
| Martins et al. 2023; Lower-body resistance training reduces interleukin-1b and transforming growth factor-b1 levels and fatigue and increases physical performance in breast cancer survivors | BCS I - III, **INT (n = 11)**: age = 52.1 ± 10.1 y, BMI = 26.2 ± 3.3, time after treatment = NR; **CON (n = 11)**: age = 57.7 ± 8.8 y, BMI = 26.2 ± 6.4, time after treatment = NR | Two-arm RCT | **RT** RT = 4 exercises, 8 to 20 repetitions at 60 to 80% 1RM for 1 to 3 sets; 3 d/w for 12 w | Stretching | 100% | After 12-h rest and fasting | IL-1β, IL-6, IL-10, TNF-α | BMI |
| Hagstrom et al. 2016; The effect of resistance training on markers of immune function and inflammation in previously sedentary women recovering from breast cancer: a randomized controlled trial | BCS I - IIIA, **INT (n = 19)**: age = 51.2 ± 8.5 y, BMI = 27.6 ± 4.2 kg/m2, time after treatment = NR; **CON (n = 15)**: age = 52.7 ± 9.4 y, BMI = 29.9 ± 6.5 kg/m2, time after treatment = NR | Two-arm RCT | **RT** RT = 8 exercises, 8 to 10 repetitions at 80% 1RM for 3 sets; 3 d/w for 16 w | Usual care | 85% | After 12-h rest and fasting | IL-6, IL-10, TNF-α, CRP | BMI |
| Sturgeon et al. 2023; WISER survivor trial: combined effect of exercise and weight loss interventions on inflammation in breast cancer survivors | BCS I - III, **INT (n = 80)**: age = 59.2 ± 8.2 y, BMI = 34.1 ± 6.3, time after treatment = ≥ 6 m; **CON (n = 81)**: age = 58.7 ± 8.4 y, BMI = 34.1 ± 5.8, time after treatment = ≥ 6 m | Two-arm RCT | **Combined AT and RT** RT = 9 exercises, 10 repetitions for 2 to 3 sets; MICT = 180 min per week; 2 d/w for 52 w | Usual care | 84% | After 12-h rest and fasting | CRP | Body weight, LM, FM |

**Legend:** INT = intervention; CON = control; RCT = randomised controlled trials; y = years; BCS = breast cancer stage; BMI = body mass index; LM = lean mass; FM = fat mass; kg = kilograms; m = months; RT = resistance training; AT = aerobic training; MICT = moderate intensity continuous training; HIIT = high intensity interval training; min = minutes; d/w = days per week; w = weeks; RM = repetition maximum; h = hours; HRmax = maximal heart rate; HRreserve = heart rate in reserve; VO2max = maximal oxygen uptake; NR = not reported.
